# Detection of Beef Adulterated with Pork Using a Low-Cost Electronic Nose Based on Colorimetric Sensors

**DOI:** 10.3390/foods9020193

**Published:** 2020-02-14

**Authors:** Fangkai Han, Xingyi Huang, Joshua H. Aheto, Dongjing Zhang, Fan Feng

**Affiliations:** 1 School of Biological and Food Engineering, Suzhou University, Bianhe Middle Road 49, Suzhou 234000, Chinazhangdongjing1987@163.com (D.Z.);; 2 School of Food and Biological Engineering, Jiangsu University, Xuefu Road 301, Zhenjiang 212013, China; kobbyaheto@yahoo.com

**Keywords:** meat adulteration, electronic nose, colorimetric sensors, chemometrics

## Abstract

The present study was aimed at developing a low-cost but rapid technique for qualitative and quantitative detection of beef adulterated with pork. An electronic nose based on colorimetric sensors was proposed. The fresh beef rib steaks and streaky pork were purchased and used from the local agricultural market in Suzhou, China. The minced beef was mixed with pork ranging at levels from 0%~100% by weight at increments of 20%. Protein, fat, and ash content were measured for validation of the differences between the pure beef and pork used in basic chemical compositions. Fisher linear discriminant analysis (Fisher LDA) and extreme learning machine (ELM) were utilized comparatively for identification of the ground pure beef, beef–pork mixtures, and pure pork. Back propagation-artificial neural network (BP-ANN) models were built for prediction of the adulteration levels. Results revealed that the ELM model built was superior to the Fisher LDA model with higher identification rates of 91.27% and 87.5% in the training and prediction sets respectively. Regarding the adulteration level prediction, the correlation coefficient and the root mean square error were 0.85 and 0.147 respectively in the prediction set of the BP-ANN model built. This suggests, from all the results, that the low-cost electronic nose based on colorimetric sensors coupled with chemometrics has a great potential in rapid detection of beef adulterated with pork.

## 1. Introduction

Meat embodies an excellent source of numerous essential nutrients for human beings. The improvement in the average incomes of many has caused an upsurge in worldwide demand for meat [1]. However, the high commercial value coupled with the increased demand for meat has attracted the attention of adulterators for several years [2].As a result, several concerns have been raised especially due to the continuous reports of adulterants in meat products that compromise their safety or quality [3,4]. Despite the risk of revenue lost through product recalls, arrest, and prosecution, somehow meat adulteration continues to attract many people. A typical case of intentional meat adulteration is the inter-species meat confounding aimed at deceiving consumers by replacing expensive meats with cheaper alternatives as is the case of adulterated pork in beef [5]. Although rarely a health hazard, this type of meat fraud violates consumer’s benefit seriously, and hampers the development of the regional meat industry gravely. Hence, it is vital to develop an effective technique for meat adulteration examination, in order to guarantee high-quality meat and meat products for the consumers.

Analytical techniques for specific proteins and DNA have been proposed for meat adulteration determination, such as chromatography, chromatography-mass spectrometry, and immunoassay, as well as electrophoretic techniques, such as polymerase chain reaction (PCR), real-time PCR, PCR-restriction fragment length polymorphism analysis, random amplification of polymorphic DNA, and amplified fragment length polymorphism. Although such techniques show a high repeatability, objectivity, and accuracy for a rapid processing situation, they tend not to be the most ideal options, owing to factors like being time-consuming, tiresome, and entailing complex laboratory protocols [6].

As one of the most active research areas in spectroscopy analytical techniques, near infrared spectroscopy (NIR) has garnered increased acceptance as a fast and non-invasive detection technique for monitoring meat quality [5,7,8]. However, owing to its single point detection technique, the spectral measurements of NIR results are generally considered as not representative enough of the whole sample to warrant being used for the determination of adulteration in meat, as different parts of the product are likely to contain different levels of the adulterant [6]. To address this limitation, spatial information is required for the generation of a distribution map. Thus, the hyperspectral imaging has been introduced to harness information from both the spectral and spatial domain simultaneously into one system, and has been applied to visualize the distribution of chemical constituents of foods [6]. However, the main drawback of the hyperspectral imaging is that it generates huge data volumes, which require long computational times, making the technique impractical for real-time online applications [9]. In addition, precision spectrometers are usually cost-intensive. Hence, it is imperative that we develop an inexpensive, rapid, and reliable analytical technique for meat adulteration detection.

Composed primarily of the non-specific chemical sensors array and a pattern recognition system, electronic nose (E-nose) was developed to mimic the human olfactory system. The gas sensors of the E-nose system are capable of detecting trace amounts of olfactory substances presented in food materials, and then produce a pattern of signals characteristic to the particular odor. When patterns from different samples are compared, differences can be correlated with perceived sample odors [10]. With the aid of chemometrics, qualitative and quantitative models could be developed using the E-nose signals to achieve food quality evaluation. Nowadays, the E-nose has been widely studied to develop a rapid analytical technique for quality prediction of the food raw materials and finished products [11,12,13,14]. Actually, an E-nose based on conductive polymer gas sensors has been successfully used for separation of pork–beef mixtures [10]. However, a major limitation associated with the use of the conductive polymers is that they are prone to ambient environmental humidity; and the sensitivities of conducting polymer films are generally an order of magnitude lower than metal oxide films [15]. Metal oxide sensors (MOSs) have also been proposed for the analysis of minced mutton mixed with pork [16,17], as well as duck adulteration in mutton [18]. In spite of the usefulness of the MOS-based E-nose, several constraints should be considered, such as high-temperature operation, high power consumption, sulfur and weak acid poisoning, limited sensor coatings, sensitive to humidity, and reduced precision [15]. Recently, the colorimetric sensor array has been introduced and applied as a solution to the afore-mentioned constraints [19].

The colorimetric sensor technique has been proven to be a powerful approach towards the detection of traces of chemical substances [20,21,22]. Various chromogenic reagents are first screened to select the most sensitive elements for the development of the colorimetric sensors for the intended usage [23].During analysis, distinct color change outlines for every individual chemical compositions can be obtained via the interactions between the measured molecules with the chromogenic reagents, through coordination reactions, acid–base interactions, dipolar reactions, π–π molecular complexation, van der Waals interactions, and physical adsorption [24,25,26,27]. With the conveniences of being simple, less costly, dependable, and easy to handle, the literature contains a number of works by researchers where artificial olfactory systems premised on colorimetric sensors have been tested for the monitoring of meat freshness or spoilage in fish [28,29], chicken [27,30], and pork [31]. However, to the best of our knowledge, the rapid detection of meat adulteration using colorimetric sensors has not been reported. On the other hand, among the popular types of livestock meat consumed in China, the average price of beef is generally the highest, and pork is the most easily obtained [3,32]. Therefore, it is of great significance to conduct research on E-nose based on the colorimetric sensor technique for qualitative and quantitative evaluation of minced beef adulterated with pork.

The present work therefore aimed to develop a rapid technique for qualitative and quantitative detection of minced beef adulterated with pork. A low-cost E-nose based on colorimetric sensors was proposed. Chemometric algorithms of Fisher linear discriminant analysis (LDA) and extreme learning machine (ELM) were applied comparatively for identification of the ground pure beef, beef–pork mixtures, and pure pork. A Back propagation-artificial neural network (BP-ANN) model was built to predict the meat adulteration levels for quantitative analysis.

## 2. Materials and Methods

### 2.1. Sample Preparation

The fresh beef rib steaks and streaky pork were purchased from retailers and certified butchers at a local agricultural market in Suzhou, China, and transported to the laboratory within 15 min at room temperature. Once in the laboratory, rib steaks (beef steaks) were sliced from the primal rib of the beef. The lean pork, on the other hand, was obtained after the pigskin, fat, and visible connective tissues of the streaky pork were discarded. Afterwards, the fresh beef and pork meat were diced and minced separately using a commercial blender (AUX-J20, Foshan Haixun electrical appliance Co., Ltd., Foshan, China). The adulteration of the minced beef was performed by mixing minced pork meat with the minced beef at levels ranging from 0%~100% (*W*/*W*) at 20% increments, and followed with mincing for 1 min. Fourteen samples were prepared for each adulteration level, each with a weight of 40.0 g. Finally, a total of 84 samples were collected for the colorimetric sensor measurements. All the meat samples were frozen and maintained in a refrigerator at 4°C before colorimetric measurements and chemical analysis.

### 2.2. General Chemical Analysis

In this work, protein, fat, and ash content were measured for validation of the differences between the pure beef and pork used in basic chemical compositions. Protein analysis was performed by the Kjeldahl determination specified in Chinese standards (GB 5009.5-2016), the fat content was estimated by the Soxhlet extraction indicated in Chinese standards (GB 5009.6-2016), and the ash content of the meat was calculated using the ignition gravimetric method described in Chinese standards (GB 5009.4-2016).

### 2.3. E-Nose Based on Colorimetric Sensors

The E-nose system constructed in this study mainly includes four parts, namely (1) the colorimetric sensor array, (2) a reaction chamber, (3) an image capturing instrument, and (4) a computer. The colorimetric sensor array was used to convert the chemical information of the volatile organic compounds (VOCs) present in the meat to the electrical signals via its color changes; the reaction chamber was used to provide an airtight space for the chromogenic agents reacted with meat VOCs. The image capturing instrument was used for acquisition of color images of the sensor array before and after exposure to the odor of sample, and the computer was used for pattern recognition and results representation.

During measurements, the sensor array was positioned in a controlled environment designated as the reaction chamber for sensing the VOCs released by the meat sample. To achieve better reaction results, it was vital to screen for appropriate chromogenic agents that would respond to the complex meat VOCs. Two fundamental requirements that ought to be met in this regard have been outlined by previous authors [22,27,28]: (1) every chemically receptive dye ought to have a center able to interact strongly with the components being measured, and (2) the center of interaction must feature strong coupling to a strong chromophore. The categories of the chemically receptive dyes that would meet these requirements are (1) Lewis acid/base dyes (i.e., porphyrins and metal ion-containing dyes), (2)Brønsted acidic or basic dyes (i.e., pH indicators), and (3) dyes with large permanent dipoles (i.e., zwitterionic solvatochromic dyes) [21]. Porphyrins and metalloporphyrins are nearly ideal for the detection of metal-ligating vapors, because of their open coordination sites for axial ligation, their large spectral shifts upon ligand binding, and their intense coloration. Common pH indicator dyes change color in response to changes in the proton (Brønsted) acidity or basicity of their environment [28].Besides, Nile red has been widely studied for sensing the low-concentration VOC benefits due to its solvatochromic properties for changing optical characteristics and its exceptional stability with regard to temperature and watervapor [33,34].

Taking into consideration the high moisture content of the fresh meat samples used in this study, twelve hydrophobic indicators were screened out and purchased from Sigma-Aldrich Chemical Co. (Shanghai, China). These indicators included

(1) manganese(3+),10,12,13,23-tetraphenyl-21H-porphyrin,trichloride;
(2) zinc-2,3,9,10,16,17,23,24-octakis-(octyloxy)-29H,31H-phthalocyanine;
(3) manganese(3+),2,12,13,15,17,18,20,23-octaethyl-21H-porphyrin,trichloride;
(4) 5,10,15,20-Tetraphenyl-21H,23H-porphinecopper;
(5) 5,10,15,20-tetraphenyl-21,22-dihydroporphyrin,zinc;
(6) [5,10,15,20-tetrakis(C6F5)-21H,23H-porphine]Fe(III) chloride;
(7) 5,10,15,20-tetraphenyl-21h,23h-porphine iron(iii) chloride;
(8) 5,10,15,20-Tetraphenyl-21H,23H-porphine Cobalt(II);
(9) 5,10,15,20-tetraphenyl-21H,23H-porphine;
(10) 2-[4-(dimethylamino)phenylazo]benzoic acid;
(11) m-Cresol, 4,4′-(3H-2,1-benzoxathiol-3-ylidene)bis(2,6-dibromo-, S,S-dioxide (8CI);
(12) 9-(Diethylamino)-5H-benzo[a]phenoxazin-5-one.

**Figure 1:**
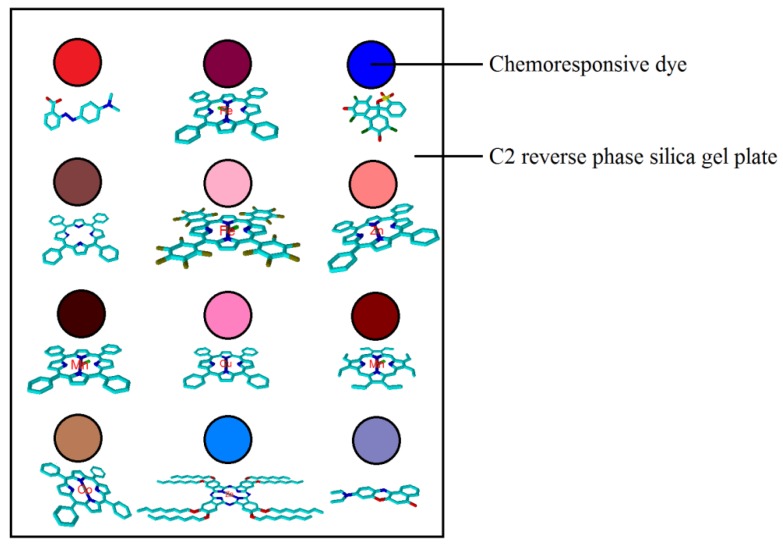
The schematic illustration of the fabricated colorimetric sensor arrays.

Afterwards, the analytically pure liquids, namely chloroform, ethanol, and acetone, purchased from Sinopharm Chemical Reagent Co. Ltd., Shanghai, China, were employed to liquidize the porphyrin compounds, pH indicators, and Nile red, respectively, to prepare the chromogenic mixtures with a concentration of 2 mg/mL. The mixtures were ultrasonicated under room temperature for 30 min. Finally, the colorimetric arrays were created by printing the chemoresponsive dyes on the reverse-phase silica gel plates (Qingdao Puke Parting Materials Co. Ltd., Qingdao, China) using 0.1 µL microcapillary pipettes. The special reverse-phase silica gel plate was selected due to the following reasons: (1) its exceptional chemical stability; (2) it has suitable surface conditions, which makes it easier for the chemodyes to spread uniformly; (3) the white surface provides a consistent background color; (4) its hydrophobic substrate is essential for reducing interference with ambient humidity; and (5) its accessible microstructure and high surface area would enhance the diffusion of the analytes to chromophore [35].After printing, the sensor arrays were quickly transferred into an itrogen-flushed glove bag before colorimetric measurements. Eventually, a colorimetric sensor array consisting of 12 chemically responsive dyes was created. The sketch map of the colorimetric sensor array developed is exhibited in Figure 1.

### 2.4. Operating Procedure and Feature Extraction

**Figure 2:**
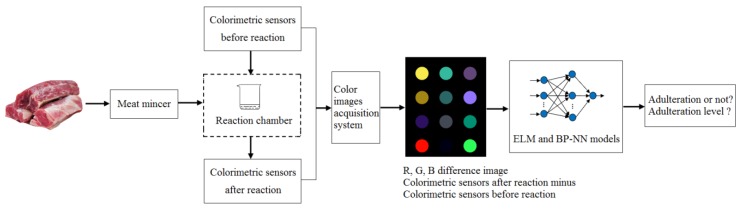
The flow chart of the measurement protocols.

A functional prototype of the E-nose system was constructed, and the flow chart is showed in Figure 2.

Each meat sample (40.0 g) was spread on the bottom of the reaction chamber (250 mL) for colorimetric measurements at room temperature. Before sensing, color images of the colorimetric sensor arrays were taken using an HP Scanjet 4890 flatbed scanner (Hewlett Packard Inc., Shanghai, China), producing red, green, and blue values at levels ranging from 0 to 255. The ability to differentiate between images was set at 400dpi by optimization. For each measurement, the constructed colorimetric sensor array was placed into the reaction chamber immediately for the dyes to react with the VOCs emitted by the meat sample. Each reaction lasted for five minutes, after which the colorimetric sensor array was taken out, scanned, and saved as the final image. The difference in images owing to color changes on the sensor array for each meat sample was obtained by subtracting the before-exposure image from the after-exposure image (i.e., red value after exposure to the VOCs of meat sample minus the red value before exposure, green minus green, blue minus blue), showed as follows [28]:

Δ*R*=|*R*_a_ − *R*_b_| Δ*G*= |*G*_a_− *G*_b_| Δ*B* = |*B*_a_− *B*_b_| (1)

where *a* represents the after reaction and *b* represents the before reaction; and Δ*R*, Δ*G*, and Δ*B* are the color-differences of red, green, and blue color channels, respectively.

In order to avoid contrived variations during measurements, the center of each sensor spot, which in the present study has a total of 450 pixels covering 12 pixel radiuses, was estimated. As a final point, a vector of the 3N-dimensional matrix (where N= 12, the number of sensors) of the color change outline was obtained.

### 2.5. Multivariate Analysis and Software

In order to develop a low-cost and rapid technique for qualitative and quantitative analysis of adulterated minced beef using colorimetric sensor-based E-nose, it is important to train calibration models by means of chemometrics for identification of the adulterated meat and prediction of the adulteration levels.

In this study, principal component analysis (PCA) was first applied on the original data matrix to transform existing correlated variables, if any, into a set of values of linearly uncorrelated variables. Then, Fisher linear discriminant analysis (Fisher LDA) and extreme learning machine (ELM) were utilized comparatively to classify the minced meat of the pure beef, beef mixed with pork, and pure pork for qualitative analysis. Furthermore, the BP-artificial neural network (BP-ANN) models were built to predict the adulteration levels for quantitative analysis.

Performances of the FisherLDA and ELM models were evaluated by the identification rate (%) shown in Equation (2).

(2) $$R=\frac{N_{1}}{N_{2}}\times 100\%$$

where *R* is the identification rate (%) in the training set or prediction set. *N*_1_ is the number of the correctly identified samples, and *N*_2_ is the number of all samples in the training set or prediction set [11].

The performance of the BP-ANN models was appraised by the root mean square error (*RMSE*) and the correlation coefficient (*r*) in the training and prediction set. The *RMSE* was calculated with following formula:

(3) $$RMSE=\sqrt{\frac{\sum_{i=1}^{n}{\left(y_{i}-{\overset{\wedge }{y}}_{i}\right)}^{2}}{n}}$$

where *n* is the number of the samples in the training set or prediction set, $y_{i}$ is the reference measurement result for the *i*th sample, and $\overset{\wedge }{y_{i}}$ is the predicted result of the model for the *i*th sample [11].

The correlation coefficients between the predicted and measured values were calculated using following formula:

(4) $$r=\sqrt{1-\frac{\sum_{i=1}^{n}{\left({\overset{\wedge }{y}}_{i}-y_{i}\right)}^{2}}{\sum_{i=1}^{n}{\left({\overset{}{y}}_{i}-\bar{y}\right)}^{2}}}$$

where: $\bar{y}$ is the mean of the references measurements results [11].

### 2.6. Software

Implementations of the algorithms used in the present work were executed with Matlab Version 7.14 (Mathworks, Natick, MA, United States) using Windows 7.

## 3. Results

### 3.1. Chemical Analysis Results

Table 1 demonstrates the differences in basic chemical compositions of the pure beef and pork meat used. It can be seen from the table that there were significant differences in protein, fat and ash content between the meat of the beef and pork used. The content of fat in the beef meat was higher than that in pork, but the content of protein and ash was lower.

### 3.2. Sensor Responses and Preprocessing

**Figure 3:**
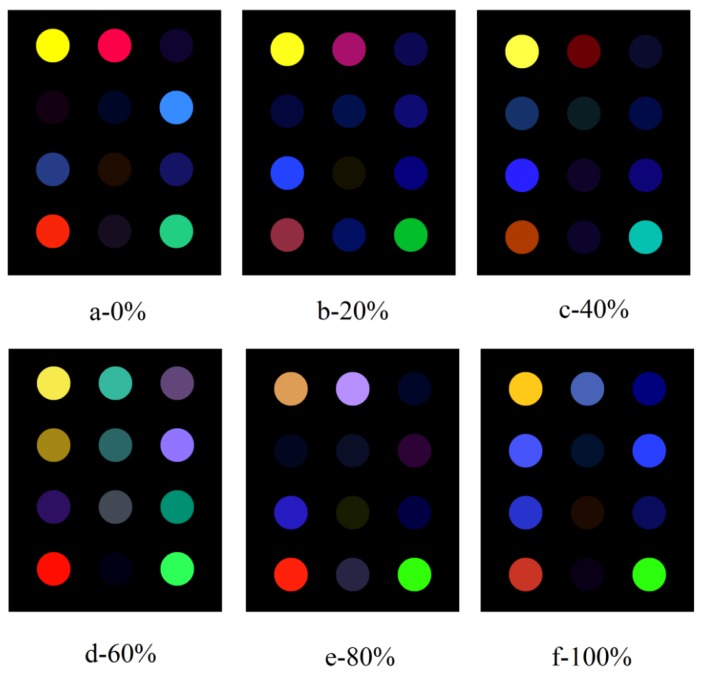
Difference images for the minced meat samples of beef adulterated with pork at different levels.

The typical difference images of the colorimetric sensor arrays before and after exposure to the minced meat samples of beef adulterated with pork at different levels is exhibited in Figure 3.

It can be seen from Figure 3 that each difference image embodies a distinct, unique, colorful fingerprint for the meat samples. Digital color information for the different images, dovetailing what is illustrated in Figure 3, is listed in Table 2. As shown in the table, even fine differences between two images could be identified, which implies that the behavior of the VOCs emitted by meat samples is related to colorific changes of the screened sensitive dyes.

The majority of identified volatiles specific to beef and pork have been extensively studied in the literature [36]. Hydrocarbons, ketones, alcohols, aldehydes, acids, esters, sulfur, and heterocyclic compounds are the main groups of VOCs released from the fresh beef and pork meat. Fat is the principal source of the specific flavor volatiles in beef and pork meats, and cooking enhances the specificity [10]. There are differences in species and concentrations of the VOCs emitted by the minced beef and pork, caused by their different general chemical compositions (see Table 1),which is the basis of the determination of beef adulterated with pork according to VOCs [10,36].

The vapor-sensitive, chemosensitive dyes (i.e., porphyrins, metalloporphyrins) employed in the present study possess accessible harmonization loci for axial ligation and exceptional coloration, and can therefore bring about molecular differentiation through the synergisms between molecules—i.e., acid–base interactions, bond development, π–π molecular complexation, and last in the sequence but equally important, van der Waals interactions and physical adsorption. Porphyrin and metalloporphyrin can display rich photophysical and electrochemical properties, and the formation of porphyrin–VOC complexes can cause VOC-dependent changes in the optical and photochemical properties of the dyes. Each pH indicator has its identifiable colorific marker related to the pH value, such that two separate pH indicator dyes in the proposed colorimetric sensor array may differ in their observable properties with the change of volatile organic acid in VOCs [37]. Besides, the positive solvatochromic properties of Nile red makes it is well suited to determine the polarities of the VOCs emitted by meat via color changes. Adhering to the afore-mentioned factors can modulate the behavior of the VOCs of interest and relate some to any color change occurring in the chemosensitive dyes (i.e., porphyrins, metalloporphyrins, pH indicators, and Nile red) on the sensor array. Therefore, the varying trends of the sensor array’s fingerprints keep in line with the meat VOCs’ varying trends [37].

In this work, to remove the collinearity of the colorimetric sensor variables and simplify the subsequent model calibrations, PCA was performed for sensor data preprocessing. PCA has been widely used for processing multidimensional and serious collinearity datasets in a multivariate problem. The new orthogonal variables, expressed by the principal components (PCs), could be achieved by determining the eigenvectors and eigenvalues of the covariance matrix of the original data matrix using PCA [38]. Each PC expresses a direct combination of the actual parameters, and these PCs explain as much as possible about the differences present in the original data set. The first principal component (PC1) expresses the largest of the variances, while the second (PC2) expresses the second-largest of the overall variations [37]. The number of PCs is less than the original variables, in the case of not losing much significant original information, so that we can decrease the influence of redundant information and simplify the later process of analyzing the problem [38].The results obtained in this study shows the cumulative contribution rate of the top nine PCs was 91.55%, which means that the top nine PCs of the colorimetric sensors characteristic variables could be used as the inputs for modeling, since they could account most of the information for the original variables. PCA results also showed that the colorimetric sensors’ data matrix that was collected exhibits obvious collinearity. This collinearity may be a result of strong, non-specific sensitivity of the dyes and wide cross-sensitivity toward the meat VOCs. In other words, each dye in the sensor array could be simultaneously sensitive to numerous volatiles, and also different dyes could be simultaneously sensitive to one of the volatiles. Therefore, this technique is not like the conventional component-by-component analyses (e.g., gas chromatography and gas chromatography-mass spectrometry), but is rather an integrated response technique towards the complex VOCs.

### 3.3. Identification of the Beef Adulterated with Pork

In the present study, Fisher LDA and ELM were applied comparatively to build models for identification of the ground pure beef, beef–pork mixtures, and the pure pork for qualitative prediction. Two-sevenths of the samples of each category were chosen, in no particular order, and designated as the training set; the remaining amounts were designated as the prediction set.

The Fisher LDA selects projection axes, defined by the discriminant functions (DFs), to fulfill the intra-class deviation formed by each intra-class projection value as little as possible, and the inter-class deviation formed by the projection value between different classes as much as possible. Afterwards, the Euclidean distance between the projection values of the unknown sample and the average variable value of each class on the DFs is calculated. Finally, the class of the unknown sample is identified according to the minimum Euclidean distance. The numbers of the DFs (*s*), sample groups (*k*), and input variables (*p*) meet the following formula:

(5) s≤min(k−1,p)

**Figure 4:**
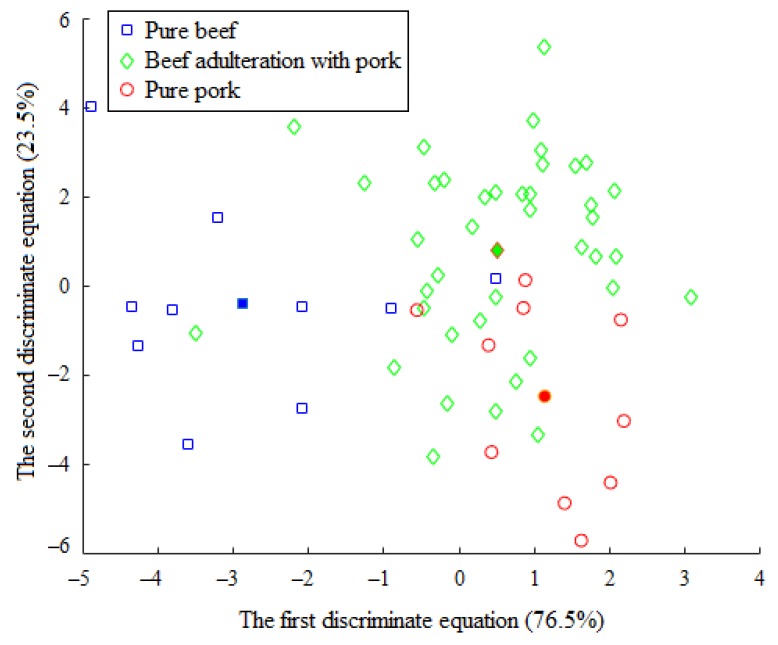
The scatter diagram of the discriminate scores of the training samples (solid markers represent the centers of the clusters).

After calculation, the contribution rate of the first and second discriminant function was 76.5% and 23.5%, respectively. Both of the DFs were utilized for prediction of the test samples. Results showed that eleven samples were misclassified, and the identification rate of the prediction set was 54.17%. Figure 4 shows the scatter diagram of the discriminate scores of the training samples. Herein, fourteen samples were classified incorrectly in the training set, and the identification rate was 76.67%. 

As shown in Figure 4, Fisher LDA gives a cluster trend for the training set. From the figure, the distance of the group centers between minced pure beef and the pure pork was farther away than the distance between each of those and the beef–pork mixtures; however, the three groups have, to a limited extent, overlapped zones. The primary cause for this overlapping may be attributed to similarity in the volatile molecules being emitted by the minced beef adulterated with pork at different levels, combined with the characteristics of non-specific and weak selectivity of the sensitive dyes. This phenomenon makes the colorimetric sensor outcomes have serious overlapping information, which leads to a very complex nonlinear relationship between the colorimetric sensors data and the sample category labels. Earlier reports suggest that with Fisher LDA, as a linear pattern classification algorithm, it is often difficult to achieve satisfactory results in dealing with nonlinear problems [39].

ELM is another novel, feed-forward neural network algorithm with the characteristics of high learning efficiency and less preset parameters. During ELM modeling, parameters of the connection weight between the input layer and hidden layer, as well as the hidden layer neuron threshold, were generated randomly. The optimal network structure of the ELM could be obtained without parameters dynamic adjustment. Theoretically, ELM is noted for providing good generalization performance at exceptionally quick computation speed [40]. Hence, it is well adapted for rapid data process in online monitoring situations. In this study, while implementing the ELM model calibration, the sigmoid function, as illustrated in Equation (6), was chosen as the activation function for the hidden layers.

(6) $$S(x)=\frac{1}{1+e^{-x}}$$

where *x* is the input variable and s (x) is the corresponding output variable.

**Figure 5:**
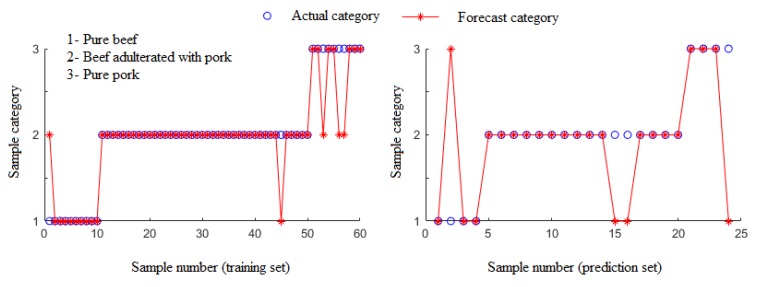
Results of training set and prediction set of extreme learning machine (ELM) model for identification of the ground pure beef, beef–pork mixtures, and the pure pork.

During ELM modeling, the neuron numbers in the hidden layer have a certain impact on the model’s performance. In the present study, the neuron numbers in the hidden layer were optimized by the minimum *RMSE* of the training and prediction set. Results showed that when the neuron number in the hidden layer was 29, the ELM model obtained the optimal prediction performance, as shown in Figure 5.

The result of the ELM model built was superior to that of Fisher LDA, with higher identification rates—i.e., 91.27% and 87.5% in the training and prediction sets, respectively. This outcome may be said to be attributed to the relationships between the data matrices, colorimetric sensor outcomes, and the category labels, being more complex than linear results from the basic principles of the colorimetric sensor technique; this technique is partially cross-sensitive to odorants, in conjunction with the good capacity for self-learning, as well as the self-adjusting property of ELM algorithm [22].

### 3.4. Prediction of Adulteration Levels

In the present study, the chemometric algorithm of BP-ANN was utilized to build a connection amongst between the colorimetric sensor dataset and the adulteration levels for quantitative determination of the minced beef adulterated with pork. BP-ANN consist of active unit arrays (artificial neurons) connected by weighted networks between the three different layers—namely, the input layer, hidden layer, and output layer—which can be successfully employed to imitate the enigmatic and entropic associations of the input and output signals. For the error between values of the prediction and expectation, BP-ANN takes a calculated error and adjusts the weights of the various layers backwards from the output layer all the way back to the input layer until a good match is achieved [38].To build the adulteration level prediction model, two-sevenths of the samples of each adulteration level was selected and designated as the training set, and the remainder was designated as the prediction set. In order to remove the dimensions and magnitudes of the input and output variables, the normalization method shown in Equation (7) was used to structure all the columns of the matrix to [0,1] [39].

(7) $$x\to y=\frac{x-x_{\min}}{x_{\max}-x_{\min}}\;x,y\in R^{n}\;x_{\min}=\min(x),x_{\max}=\max(x)$$

where *x* is the original variable, *y* is the transformed variable.

**Figure 6:**
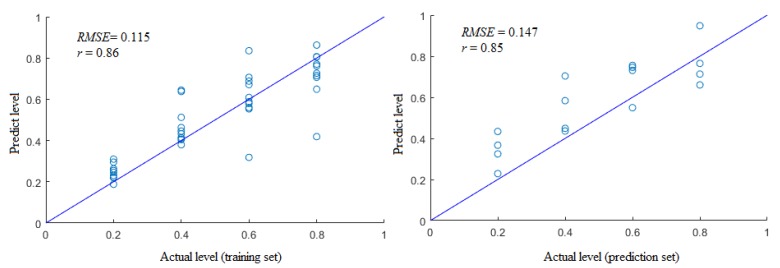
The root mean square error (*RMSE*) and correlation coefficient (*r*) of the BP-artificial neural network (BP-ANN) model for adulteration level prediction.

In the development of the BP-ANN model, after a few endeavors to improve the outputs, both the learning rate factor and momentum factor were set at 0.1, the scale function was the “tan h” function, the allowable training error was 0.0002, and the maximal times of training were 10.000. The concealed nodes were improved based on“trial and error”, and ideal hidden nodes were determined by the minimal *RMSE* value [38,41]. Finally, the optimum network architecture was obtained with topological architecture 9−5−1. The *RMSE* and *r* of the BP-ANN model built for the adulteration level prediction are shown in Figure 6.

In this study, the correlation output found between the training and prediction sets of the gas sensors (i.e., color change) and the adulteration levels of the beef-pork mixtures was found to be good, which was mainly due by BP-ANN having a great advantage in processing nonlinear problems.

## 4. Conclusions

The present study was set up to develop a low-cost and rapid technique for analysis of the beef adulterated with pork. The E-nose setup premised on chemosensitive dyes in conjunction with chemometrics was proposed. For identification of pure ground beef, beef-pork mixtures, and pure pork, an ELM model built that was better than that of Fisher LDA, with higher identification rates of 91.27% and 87.5% in the training and prediction set, respectively. Good correlations between the gas sensor results (i.e., color change) and the adulteration levels of the beef–pork mixtures were obtained in the training and prediction set of BP-ANN model. All findings suggest that the low-cost E-nose fabricated with chemosensitive dyes coupled with chemometrics has great potential for the rapid detection of minced beef adulterated with pork.

## Figures and Tables

**Table 1:** Chemical indices of the pure beef and pork meat used (g/100g).

	Crude Protein	Total Lipid	Total Ash
beef	14 ± 0.85^a^	34 ± 4.0^a^	0.62 ± 0.10^a^
pork	18 ± 3.0^b^	4.3 ± 0.47^b^	1.0 ± 0.19^b^

Values (*n*= 12) are presented as mean ± standard deviation. Values in the same column with different superscripts were significantly different (*p* < 0.05).

**Table 2:** Digital color information of the difference images for the minced meat samples of beef adulterated with pork at different levels.

Adulteration Level	20%			40%			60%			80%		
	C1 in Figure 3b	C2 in Figure 3b	C3 in Figure 3b	C1 in Figure 3c	C2 in Figure 3c	C3 in Figure 3c	C1 in Figure 3d	C2 in Figure 3d	C3 in Figure 3d	C1 in Figure 3e	C2 in Figure 3e	C3 in Figure 3e
Red	7.2	4.8	0.49	12	5.1	0.55	7.9	2.0	3.4	8.1	6.8	0.031
	0.25	0.21	0.54	1.1	0.50	0.053	5.4	1.7	4.8	0.089	0.40	1.7
	1.2	0.70	0.31	2.0	0.79	0.65	1.7	2.4	0.37	1.4	0.87	0.068
	4.2	0.15	0.22	8.4	0.62	0.31	8.2	0.35	1.8	9.3	1.4	1.8
Green	7.3	0.49	0.30	11	0.024	0.53	9.3	7.4	3.0	6.7	6.1	0.33
	0.2	0.53	0.34	2.2	1.3	0.52	5.4	4.2	4.7	0.40	0.66	0.19
	1.9	0.53	0.04	1.5	0.22	0.20	0.86	3.1	5.8	1.2	1.2	0.090
	1.3	0.47	5.4	2.6	0.18	8.6	0.74	0.25	10	1.5	1.6	111
Blue	0.18	0.56	0.44	1.7	0.11	1.1	3.7	7.6	5.8	2.3	6.8	1.1
	0.34	0.41	0.60	2.6	0.89	1.8	0.98	4.9	12	0.86	1.1	1.4
	1.3	0.05	0.64	6.3	1.0	3.0	4.7	4.0	5.5	5.1	0.049	1.8
	0.36	0.51	0.25	0.022	1.1	4.4	0.024	1.0	4.2	0.30	1.8	0.31

C: column.

## References

[1] Sanchez-Sabate R., Sabaté J. (2019). Consumer attitudes towards environmental concerns of meat consumption: A systematic review. Int. J. Environ. Res. Public Health.

[2] Barai B., Nayak R., Singhal R., Kulkarni P. (1992). Approaches to the detection of meat adulteration. Trends Food Sci. Technol..

[3] Song Q., Chen Y., Zhao L., Ouyang H., Song J. (2019). Monitoring of sausage products sold in Sichuan Province, China: A first comprehensive report on meat species’ authenticity determination. Sci. Rep..

[4] Li X., Gao X., Guan Y. (2019). A Novel Isothermal Amplification Method for Detecting Mouse Source Component in Meat. J. AOAC Int..

[5] Alamprese C., Amigo J.M., Casiraghi E., Engelsen S.B. (2016). Identification and quantification of turkey meat adulteration in fresh, frozen-thawed and cooked minced beef by FT-NIR spectroscopy and chemometrics. Meat Sci..

[6] Kamruzzaman M., Makino Y., Oshita S., Liu S. (2015). Assessment of visible near-infrared hyperspectral imaging as a tool for detection of horsemeat adulteration in minced beef. Food Bioprocess Technol..

[7] Schmutzler M., Beganovic A., Böhler G., Huck C.W. (2015). Methods for detection of pork adulteration in veal product based on FT-NIR spectroscopy for laboratory, industrial and on-site analysis. Food Control.

[8] López-Maestresalas A., Insausti K., Jarén C., Pérez-Roncal C., Urrutia O., Beriain M.J., Arazuri S. (2019). Detection of minced lamb and beef fraud using NIR spectroscopy. Food Control.

[9] Erkinbaev C., Henderson K., Paliwal J. (2017). Discrimination of gluten-free oats from contaminants using near infrared hyperspectral imaging technique. Food Control.

[10] Turhan M., Balaban M.O., Nazan Turhan K., Luzuriaga D. (1998). Potential use of electronic nose technique for detection of meat adulteration: Separation of pork-beef mixtures. Fleischwirtsch. Int..

[11] Han F., Huang X., Teye E., Gu F., Gu H. (2014). Nondestructive detection of fish freshness during its preservation by combining electronic nose and electronic tongue techniques in conjunction with chemometric analysis. Anal. Methods.

[12] Baietto M., Wilson A.D. (2015). Electronic-nose applications for fruit identification, ripeness and quality grading. Sensors.

[13] Di Rosa A.R., Leone F., Cheli F., Chiofalo V. (2017). Fusion of electronic nose, electronic tongue and computer vision for animal source food authentication and quality assessment—A review. J. Food Eng..

[14] Liu Q., Zhao N., Zhou D., Sun Y., Sun K., Pan L., Tu K. (2018). Discrimination and growth tracking of fungi contamination in peaches using electronic nose. Food Chem..

[15] Wilson A.D., Baietto M. (2009). Applications and advances in electronic-nose technologies. Sensors.

[16] Tian X., Wang J., Cui S. (2013). Analysis of pork adulteration in minced mutton using electronic nose of metal oxide sensors. J. Food Eng..

[17] Tian X., Wang J., Ma Z., Li M., Wei Z. (2019). Combination of an E-Nose and an E-Tongue for Adulteration Detection of Minced Mutton Mixed with Pork. J. Food Qual..

[18] Wang Q., Li L., Ding W., Zhang D., Wang J., Reed K., Zhang B. (2019). Adulterant identification in mutton by electronic nose and gas chromatography-mass spectrometer. Food Control.

[19] Kutsanedzie F.Y., Guo Z., Chen Q. (2019). Advances in Nondestructive Methods for Meat Quality and Safety Monitoring. Food Rev. Int..

[20] Rakow N.A., Suslick K.S. (2000). A colorimetric sensor array for odour visualization. Nature.

[21] Suslick K.S., Rakow N.A., Sen A. (2004). Colorimetric sensor arrays for molecular recognition. Tetrahedron.

[22] Han F., Huang X., Teye E. (2019). Novel prediction of heavy metal residues in fish using a low-cost optical electronic tongue system based on colorimetric sensors array. J. Food Process Eng..

[23] Xiaowei H., Xiaobo Z., Jiewen Z., Jiyong S., Zhihua L., Tingting S. (2015). Monitoring the biogenic amines in Chinese traditional salted pork in jelly (Yao-meat) by colorimetric sensor array based on nine natural pigments. Int. J. Food Sci. Technol..

[24] Zhang C., Bailey D.P., Suslick K.S. (2006). Colorimetric sensor arrays for the analysis of beers: A feasibility study. J. Agric. Food Chem..

[25] Bang J.H., Lim S.H., Park E., Suslick K.S. (2008). Chemically responsive nanoporous pigments: Colorimetric sensor arrays and the identification of aliphatic amines. Langmuir.

[26] Lim S.H., Musto C.J., Park E., Zhong W., Suslick K.S. (2008). A colorimetric sensor array for detection and identification of sugars. Org. Lett..

[27] Chen Q., Hu W., Su J., Li H., Ouyang Q., Zhao J. (2016). Nondestructively sensing of total viable count (TVC) in chicken using an artificial olfaction system based colorimetric sensor array. J. Food Eng..

[28] Huang X., Xin J., Zhao J. (2011). A novel technique for rapid evaluation of fish freshness using colorimetric sensor array. J. Food Eng..

[29] Zaragozá P., Fuentes A., Ruiz-Rico M., Vivancos J.-L., Fernández-Segovia I., Ros-Lis J.V., Barat J.M., Martínez-Máñez R. (2015). Development of a colorimetric sensor array for squid spoilage assessment. Food Chem..

[30] Salinas Y., Ros-Lis J.V., Vivancos J.-L., Martínez-Máñez R., Marcos M.D., Aucejo S., Herranz N., Lorente I. (2012). Monitoring of chicken meat freshness by means of a colorimetric sensor array. Analyst.

[31] Salinas Y., Ros-Lis J.V., Vivancos J.-L., Martínez-Máñez R., Marcos M.D., Aucejo S., Herranz N., Lorente I., Garcia E. (2014). A novel colorimetric sensor array for monitoring fresh pork sausages spoilage. Food Control.

[32] Zhu A., Zhi W., Qiu Y., Wei L., Tian J., Pan Z., Kang X., Gu W., Duan L. (2019). Surveillance study of the prevalence and antimicrobial resistance of Salmonella in pork from open markets in Xuzhou, China. Food Control.

[33] Khan M.R.R., Kang B.-H., Yeom S.-H., Kwon D.-H., Kang S.-W. (2013). Fiber-optic pulse width modulation sensor for low concentration VOC gas. Sens. Actuators B Chem..

[34] Chen Y., Fu G., Zilberman Y., Ruan W., Ameri S.K., Zhang Y.S., Miller E., Sonkusale S.R. (2017). Low cost smart phone diagnostics for food using paper-based colorimetric sensor arrays. Food Control.

[35] Xiao-wei H., Xiao-bo Z., Ji-yong S., Zhi-hua L., Jie-wen Z. (2018). Colorimetric sensor arrays based on chemo-responsive dyes for food odor visualization. Trends Food Sci. Technol..

[36] Shahidi F., Rubin L.J., D’Souza L.A., Teranishi R., Buttery R.G. (1986). Meat flavor volatiles: A review of the composition, techniques of analysis, and sensory evaluation. Crit. Rev. Food Sci. Nutr..

[37] Chen Q., Liu A., Zhao J., Ouyang Q., Sun Z., Huang L. (2013). Monitoring vinegar acetic fermentation using a colorimetric sensor array. Sens. Actuators B Chem..

[38] Han F., Huang X., Teye E., Gu H. (2015). Quantitative analysis of fish microbiological quality using electronic tongue coupled with nonlinear pattern recognition algorithms. J. Food Saf..

[39] Han F., Huang X., Teye E., Gu H., Dai H., Yao L. (2014). A nondestructive method for fish freshness determination with electronic tongue combined with linear and non-linear multivariate algorithms. Czech J. Food Sci..

[40] Huang G.-B., Zhu Q.-Y., Siew C.-K. (2006). Extreme learning machine: Theory and applications. Neurocomputing.

[41] Lin H., Chen Q., Zhao J., Zhou P. (2009). Determination of free amino acid content in Radix Pseudostellariae using near infrared (NIR) spectroscopy and different multivariate calibrations. J. Pharm. Biomed. Anal..

